# Specificity of CD8^+^ T-Cell Responses Following Vaccination with Conserved Regions of HIV-1 in Nairobi, Kenya

**DOI:** 10.3390/vaccines8020260

**Published:** 2020-05-29

**Authors:** Yehia S. Mohamed, Nicola J. Borthwick, Nathifa Moyo, Hayato Murakoshi, Tomohiro Akahoshi, Francesca Siliquini, Zara Hannoun, Alison Crook, Peter Hayes, Patricia E. Fast, Gaudensia Mutua, Walter Jaoko, Sandra Silva-Arrieta, Anuska Llano, Christian Brander, Masafumi Takiguchi, Tomáš Hanke

**Affiliations:** 1 The Jenner Institute, University of Oxford, Oxford OX3 7DQ, UK; yehiasmohamed@hotmail.com (Y.S.M.); nicola.borthwick@ndm.ox.ac.uk (N.J.B.); nathifa.moyo@ndm.ox.ac.uk (N.M.); francesca.siliquini@studio.unibo.it (F.S.); zhannoun@gmail.com (Z.H.); alison.crook@ndm.ox.ac.uk (A.C.); 2 Department of Microbiology and Immunology, Faculty of Pharmacy, Al-Azhar University, Cairo 11823, Egypt; 3 Joint Research Center for Human Retrovirus Infection, Kumamoto University, Kumamoto 860-0811, Japan; tlmura@kumamoto-u.ac.jp (H.M.); tlakahoshi@gmail.com (T.A.); masafumi@kumamoto-u.ac.jp (M.T.); 4 International AIDS Vaccine Initiative IAVI-Human Immunology Laboratory, Imperial College London, London SW10 9NH, UK; p.hayes@imperial.ac.uk; 5 International AIDS Vaccine Initiative-New York, New York, NY 10004, USA; pfast@iavi.org; 6 KAVI-Institute of Clinical Research, University of Nairobi, Nairobi 19676 00202, Kenya; gmutua@kaviuon.org (G.M.); wjaoko@kaviuon.org (W.J.); 7 IrsiCaixa AIDS Research Institute-HIVACAT, Hospital Universitari Germans Trias i Pujol, 08916 Barcelona, Spain; ssilva@irsicaixa.es (S.S.-A.); ALlano@irsicaixa.es (A.L.); cbrander@irsicaixa.es (C.B.); 8 Faculty of Medicine, Universitat de Vic-Central de Catalunya (UVic-UCC), 08500 Vic, Spain; 9 Catalan Institution for Research and Advanced Studies (ICREA), 08010 Barcelona, Spain

**Keywords:** HIV vaccine, HIVconsv, conserved regions, CD8 epitopes, HLA class I epitopes, T cell vaccine, African HLA

## Abstract

Sub-Saharan Africa carries the biggest burden of the human immunodeficiency virus type 1 (HIV-1)/AIDS epidemic and is in an urgent need of an effective vaccine. CD8^+^ T cells are an important component of the host immune response to HIV-1 and may need to be harnessed if a vaccine is to be effective. CD8^+^ T cells recognize human leukocyte antigen (HLA)-associated viral epitopes and the HLA alleles vary significantly among different ethnic groups. It follows that definition of HIV-1-derived peptides recognized by CD8^+^ T cells in the geographically relevant regions will critically guide vaccine development. Here, we study fine details of CD8^+^ T-cell responses elicited in HIV-1/2-uninfected individuals in Nairobi, Kenya, who received a candidate vaccine delivering conserved regions of HIV-1 proteins called HIVconsv. Using 10-day cell lines established by in vitro peptide restimulation of cryopreserved PBMC and stably HLA-transfected 721.221/C1R cell lines, we confirm experimentally many already defined epitopes, for a number of epitopes we define the restricting HLA molecule(s) and describe four novel HLA-epitope pairs. We also identify specific dominance patterns, a promiscuous T-cell epitope and a rescue of suboptimal T-cell epitope induction in vivo by its functional variant, which all together inform vaccine design.

## 1. Introduction

Human leukocyte antigen (HLA) molecules are highly polymorphic cell surface glycoproteins, which bind and present processed antigenic peptides derived from viruses, such as human immunodeficiency virus type 1 (HIV-1) to the cells of the immune system [1,2]. Cytotoxic T lymphocytes (CTL) recognize peptide-loaded HLA class I through their T-cell receptor (TCR) and trigger apoptotic pathways in virus-infected cells serving as virus factories, thereby limit production of new infectious virions [3,4]. Polymorphisms in HLA class I alleles are generally clustered within the sequences encoding the HLA peptide-binding groove, which selects for a range of bound peptide specificities that determine the successfully processed, host-cell presented peptidome. This finite set of epitopes combined with the host’s TCR repertoire largely determines whether or not the host can mount an effective T-cell response [5,6]. Viruses with highly variable genomes such as HIV-1 rapidly mutate epitopes to escape T-cell recognition and the immune pressure selects the fittest escaped variants to overgrow [7,8,9,10]. This immediately suggests that epitopes that are easily escaped with minimal fitness cost are less protective than epitopes within functionally constraint, and therefore conserved protein regions [11,12,13,14,15]. HLA haplotype, epitope specificity of the immune responses and escape in targeted epitopes are among the major factors determining the rate of disease progression in untreated HIV-1 infection [16,17,18,19]. Despite the extensive HLA polymorphism and based on peptide binding preferences, many HLA class I alleles can be clustered into 9 supertypes with common structural features and overlapping associated peptidomes [18,20,21,22]. On the population level, common HLAs/HLA supertypes are theoretically less protective relative to the rare ones due to the increased probability of transmission of already escaped viruses; however, through selection of resistant individuals, rare HLAs will eventually become more common and, therefore, less protective [12,22,23,24,25,26]. Thus, hosts with their HLA haplotypes and associated peptidomes are constantly shaped by much faster evolving infectious agents and other diseases.

T cells clearly play a protective role in HIV-1 infection [27,28,29,30,31] and HIV-1 T-cell vaccine development would be greatly facilitated by a definition of functional correlates of T-cell protection [32,33,34,35,36] including cumulative identification of protective epitopes in vulnerable HIV-1 sites [37,38,39,40,41,42,43]. The current strategies for vaccine development are likely biased towards inclusion of immunodominant epitopes restricted by most frequent/studied HLA class I alleles, and HLAs associated with a slow disease progression [14,44,45,46,47,48,49]. In contrast, our working hypothesis postulates that focusing vaccine-elicited T cells on the functionally conserved regions of HIV-1, which are common to most global variants and are hard to mutate, will be effective in slowing and controlling HIV-1 infection [41,42,43,50,51,52,53,54,55]. These regions contain epitopes typically subdominant in natural HIV-1 infection, but are capable of inducting robust T-cell responses when delivered by a potent vaccination regimen [50,56,57,58,59,60]. Such vaccine-elicited responses thus represent a rich source of previously undescribed, potentially important epitopes [61,62,63]. In the present work, we characterize CD8^+^ T-cell responses induced by the first generation of conserved-region vaccines expressing immunogen HIVconsv [53] in an HIV-1-negative population in the Kangemi district of Nairobi, Kenya [60], and confirm a number of already-known as well as identify novel conserved CD8^+^ T-cell epitopes. These results are discussed in the context of development of an effective anti-HIV-1 T-cell vaccine.

## 2. Materials and Methods

### 2.1. Trial HIV-CORE 004

The HIV-CORE 004 trial was conducted at the KAVI-Institute for Clinical Studies (KAVI-ICR), Kangemi site, Nairobi, Kenya between April 2014 and August 2015 and recruited healthy adults of low risk of HIV-1 infection. It had all the appropriate ethics committee and regulatory approvals and was conducted according to the principles of the Declaration of Helsinki (2008) and complied with the International Conference on Harmonization Good Clinical Practice guidelines as reported previously [60]. The trial is registered on the Pan African Clinical Trials Registry under reference PACTR201403000794397 and its ClinicalTrials.gov Identifier is NCT02099994.

### 2.2. Cryopreserved PBMC Samples

The PBMC used in this study were cryopreserved samples from HIV-CORE 004 and subsequently held under Human Tissue Act license 12217 at The Jenner Institute, University of Oxford, Oxford, UK. For epitope mapping, the samples used were from peak responses or the nearest available sample (between 2 and 12 weeks after vaccination).

### 2.3. Peptides

HIVconsv-matched 15-mer peptides overlapping by 11 amino acids (aa) >80% pure (Ana Spec, San Jose, CA, USA, 95131) and their shorter derivatives (Synpeptide, Shanghai, China) were reconstituted to 10-40 mg/mL in dimethyl sulfoxide (DMSO) (Sigma Aldridge, Pool, UK) and diluted to working stock solutions of 4 mg/mL in PBS as described previously [50].

### 2.4. HLA-Transfected Cell Lines

Open-reading frames coding for HLA-A*03:01, HLA-B*07:05 and HLA-B*57:03 were amplified with BamHI-Kozak-sequence and XbaI sites at their 5′- and 3′-ends, respectively, by PCR using cDNA synthesized from RNA of HLA-positive donors. The PCR products were cloned using TOPO TA or Zero Blunt Topo cloning kit (Invitrogen, Carlsbad, CA, USA). The BamHI-XbaI 1.1-kbp fragment were inserted into the same sites of the pcDNA3.1/Neo^+^ expression plasmid (Invitrogen, Carlsbad, CA, USA), which was used to generate 721.221-CD4 cells stably expressing HLA-A*03:01, HLA-B*07:05, or HLA-B*57:03. C1R cells expressing HLA-B*15:01 [64] and HLA-B*53:01 [65] were generated previously.

### 2.5. Cultured IFN-γ ELISPOT Assay

For epitope mapping, short-term cell lines (STCL) were generated from PBMC cultured in vitro for 10 days with peptide pools containing 30–33 peptides at 1.5 µg/mL per peptide in R10 culture medium (RPMI 1460 supplemented with 10% FBS, 2 mM L-glutamine, 1 mM sodium pyruvate, 10 mM HEPES, and penicillin–streptomycin antibiotics (Sigma-Aldrich, St. Louis, MO, USA)). Interleukin (IL)-7 at 25 ng/mL (Peprotech, London, UK.) was added at the start of the culture and IL-2 at 100 IU/mL was added on days 3 and 7. After 10 days, the cells were washed and rested in medium without IL-2 (R&D Systems) for at least 24 h. For the ELISPOT assay, each 15-mer peptide was tested in duplicate and the assay performed using 4.0 × 10^4^ cells per well as described previously 3.

### 2.6. Intracellular Cytokine Staining (ICS) Assay and HLA Restriction

STCL were generated as above using a single “parental” 15-mer peptide. To examine optimal peptide length and their HLA restriction, 721.221 or C1R cells transfected with a single HLA class I allele were pulsed with short peptides and used as antigen-presenting cells for stimulation of STCL effectors in an ICS assay, which also confirmed that the responding T cells were CD8^+^ [62]. For tetramer reactivity, 10^6^ STCL cells were stained with pre-titrated amounts of PE-conjugated tetramer (NIH Tetramer Facility, Emory University Vaccine Center, Atlanta, GA, USA) in FACS tubes at room temperature for 10 min. For both HLA and tetramer procedures, a mix of anti-CD8 FITC, anti-CD4 PE, anti-CD3 PE-CF594, anti-IFN-γ V450 (BD Biosciences, Wokingham, UK), and anti-CD3 ECD (Beckman-Coulter, High Wycombe, UK) monoclonal antibodies (mAbs), and LIVE/DEAD fixable cell stain Aqua were added and the tubes incubated for a further 20 min at room temperature. The cells were washed with FACS buffer, fixed with 1% paraformaldehyde prior to analysis, acquired by a Fortessa flow cytometer (Becton-Dickinson, Franklin lakes, NJ, USA) and analyzed using the FlowJo software (Tree Star).

### 2.7. In Vitro Assay for Peptide-HLA-Class I Complex Formation

The interaction of some peptides with HLA-A*02:01 was validated using an easYmer kit (Immune Aware Aps, Virum, Denmark) following the manufacturer’s instructions. This kit provided peptide-receptive and biotinylated HLA-A*02:01 heavy chains and peptide NLVPMVATV as a positive control. The assay captures refolded HLA class I complexes on streptavidin-coated microbeads (Spherotech, Saxon Europe, Kelso, UK) and quantifies them using PE-conjugated anti-β_2_-microglobulin (Insight Biotechnology, Wembley, UK).

### 2.8. Cytotoxicity Assay

A flow cytometry-based assay was used to measure the cytotoxic T lymphocyte (CTL) effector function of the YV9 clone [61]. For the CTL assay, autologous B-lymphoblastoid cell lines (B-LCL), were the targets. The Cell Trace 5 (6)-carboxyfluorescein diacetate N-succinimidyl ester (CFSE) (Molecular Probes; Invitrogen, Carlsbad, CA, USA) -labelled cells pulsed with peptide at concentrations ranging 0.01–10.0 µM or unpulsed were mixed with Far Red-labelled BCL and the CD8^+^ clone at 10^5^ cells at 1:1:1 ratio in a round-bottom microtiter plate. After culture overnight, the cells were washed and stained with LIVE/DEAD Aqua (Thermo Fisher, Waltham, MA, USA) and fluorescence measured using an LSRII flow cytometer (Becton-Dickinson, Franklin lakes, NJ, USA). The ratio of CFSE: Far Red labelled cells in the absence (E^o^) and presence (E^+^) of effector CD8^+^ T cells was used to measure the cytotoxicity using the equation: % Lysis = E^o^ − E^+^/E^o^ × 100.

## 3. Results

### 3.1. The Study Subjects and Vaccination

**Figure 1:**
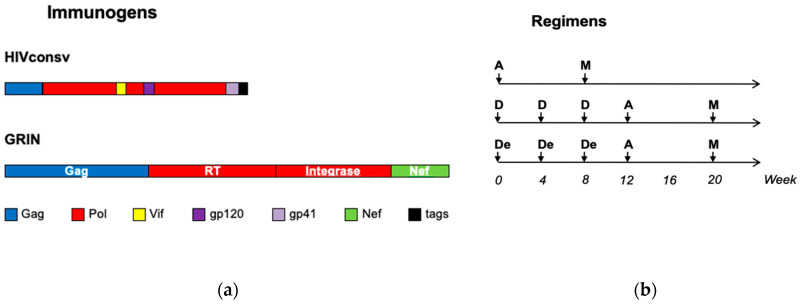
The HIV-CORE 004 trial: Vaccine immunogens and regimens. (**a**) Schematic representation of the HIVconsv immunogen derived from 14 conserved regions of the human immunodeficiency virus type 1 (HIV-1) proteome. For each segment, the clade of consensus aa sequence is shown above and the HIV-1 proteins from which it was derived are color-coded. C-terminal CD8^+^ T-cell and monoclonal antibody epitopes (tags) were added to facilitate preclinical vaccine development and manufacture. The GRIN (Gag-RT-Integrase-Nef) immunogen matched the HIVconsv sequence in 604 out of 776 aa (78%) and these 604 aa of GRIN had 97.6% homology with the HIVconsv protein. The two immunogens are drawn approximately to scale. (**b**) Three vaccination regimens tested in this trial employed A for Ad35-GRIN—replication-deficient engineered human adenovirus serotype 35 expressing GRIN at 5 × 10^10^ virus particles; M for MVA.HIVconsv—replication-deficient poxvirus expressing HIVconsv at 2 × 10^8^ plaque-forming units; and D or De for SG2.HIVconsv—”naked” plasmid DNA expressing HIVconsv at 4 mg delivered without or with electroporation, respectively. In vivo intramuscular electroporation was carried out using the TriGrid Delivery System of Ichor Medical Systems, San Diego, CA, USA. Vaccines were delivered by intramuscular needle injection.

Healthy, HIV-1/2-negative adults in Nairobi, Kenya recruited into trial HIV-CORE 004 received a combination of HIVconsv and GRIN candidate HIV-1 vaccines as described [60]. The HIVconsv immunogen was assembled from conserved regions of HIV-1 proteins and its gene was delivered by a plasmid DNA (pSG2.HIVconsv or D) and replication-deficient poxvirus modified vaccinia virus Ankara (MVA.HIVconsv or M) [50,53]. The GRIN immunogen was a fusion of full-length Gag, Reverse Transcriptase (RT), Integrase and Nef, and was delivered by replication-deficient human adenovirus serotype 35 (Ad35-GRIN or A) [66,67]. Ad35-GRIN substituted for the simian adenovirus serotype 63-vectored HIVconsv vaccine used in previous trials [50,56,57,58,59], to which we lost freedom to operate following GlaxoSmithKline acquisition of the ChAdV63 vaccine vector. GRIN had an extensive sequence match to HIVconsv as GRIN covered almost 80% of all HIVconsv (Figure 1) [60].

### 3.2. HLA Allele Frequency in Nairobi Volunteers

**Figure 2:**
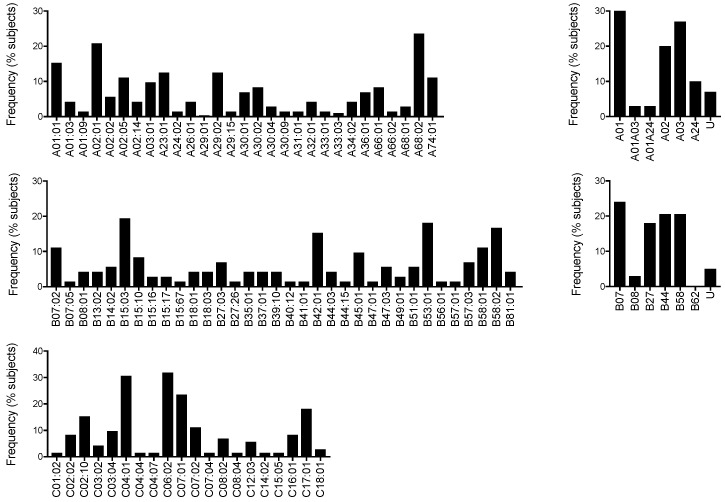
Human leukocyte antigen (HLA) class I alleles among Nairobi volunteers. HLA allele (**left**) and supertypes (**right**) frequencies are shown (*n* = 72). U—unclassified supertype.

Trial HIV-CORE 004 recruited 72 healthy, HIV-1/2-negative individuals, whose HLA class I A, B, and C alleles, the regimen they received and their total peak fresh ex vivo IFN-γ ELISPOT assay magnitude of HIVconsv-specific T cells are shown in Table S1. The HLA genotype (Figure 2, left) and HLA supertype (Figure 2, right) frequencies were determined and the most frequent alleles in this cohort for the three loci were found to be HLA-A*68:02 and A*02:01, HLA-B*15:03 and B*53:01, and HLA-C*06:02 and C*04:01, respectively, out of a total of 83 alleles (29A, 34B, and 20C).

### 3.3. Definition of Optimal CD8^+^ T-Cell Epitopes Restricted by African HLAs

**Figure 3:**
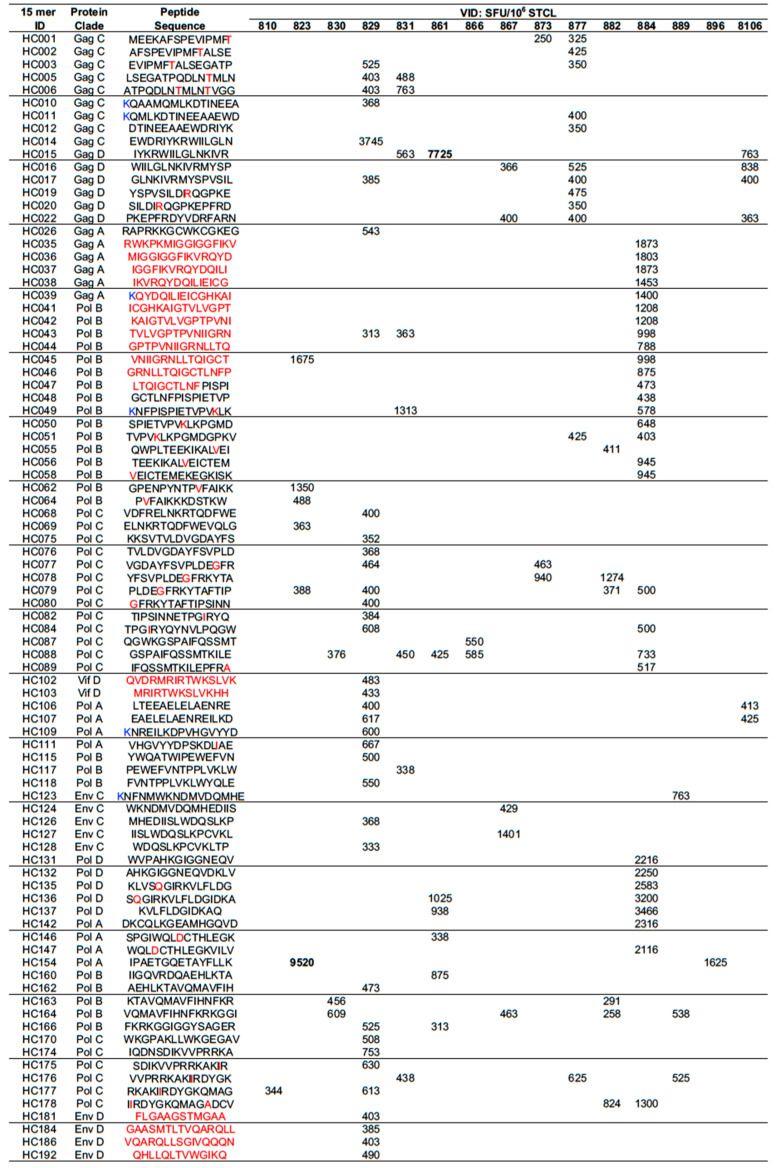
Mapping of stimulatory 15-mer peptides. PBMCs were expanded in vitro with 1 of 6 pools of overlapping 15/11 aa peptides spanning the entire HIVconsv immunogen for 10 days to establish short-term cell lines (STCL) and tested in an IFN-γ ELISPOT assay against individual peptides. The frequencies of responding IFN-γ SFU stimulated by peptides are indicated. Peptides and aa shown in red were present in HIVconsv and absent from GRIN. Terminal ‘K’ (blue) were added for solubility (non-HIV). VID—volunteer identification.

The primary trial readout used 6 pools of 15-mer peptides overlapping by 11 aa (15/11) spanning the entire length of the HIVconsv immunogen in a fresh ex vivo IFN-γ ELISPOT assay. As previously reported, the AM, DDDAM and DeDeDeAM regimens induced median frequencies of 2158, 3590, and 2369 spot-forming unit (SFU)/10^6^ PBMC, respectively, of HIVconsv-specific T cells, which recognized median of 6 out of 6 peptide pools [60]. Here, the primary aim was to define the specificity rather than magnitude of vaccine-elicited T cells for frequent African HLA alleles. PBMC were first re-stimulated in vitro with individual previously identified, strongly positive peptide pools for 10 days to generate STCL. These STCL were then tested against individual 15-mer peptides contained in the pool in an IFN-γ ELISPOT assay. Many peptides were recognized by more than one individual and the most frequently targeted peptides were contained in both the HIVconsv and GRIN immunogen sequences (Figure 3). In the second stage, STCL were expanded from PBMC using individual “parental” 15-mers and assayed against progressively shorter peptides in a polychromatic intracellular cytokine staining (ICS) assay. The HLA restriction of optimal peptides was confirmed by IFN-γ production by CD3^+^CD8^+^ cells of the STCL using peptide-pulsed B-LCL, 721.221 and/or C1R cell lines transfected with a single HLA-class I molecule (see Figure S1 for gating strategy) and reactivity with HLA-peptide tetrameric complexes. Results are presented in the order of the parental 15-mer overlapping peptides in Figures S2–S8 and summarized in Table 1. Overall, we newly defined and/or confirmed 21 optimal-length CD8^+^ T cell-stimulatory epitopes and 8 HLA-epitope pairs, of which 4 pairs have not been reported previously.

### 3.4. Peptide FF9 Stimulates Functional T-Cell Response via Three HLA Supertypes

**Figure 4:**
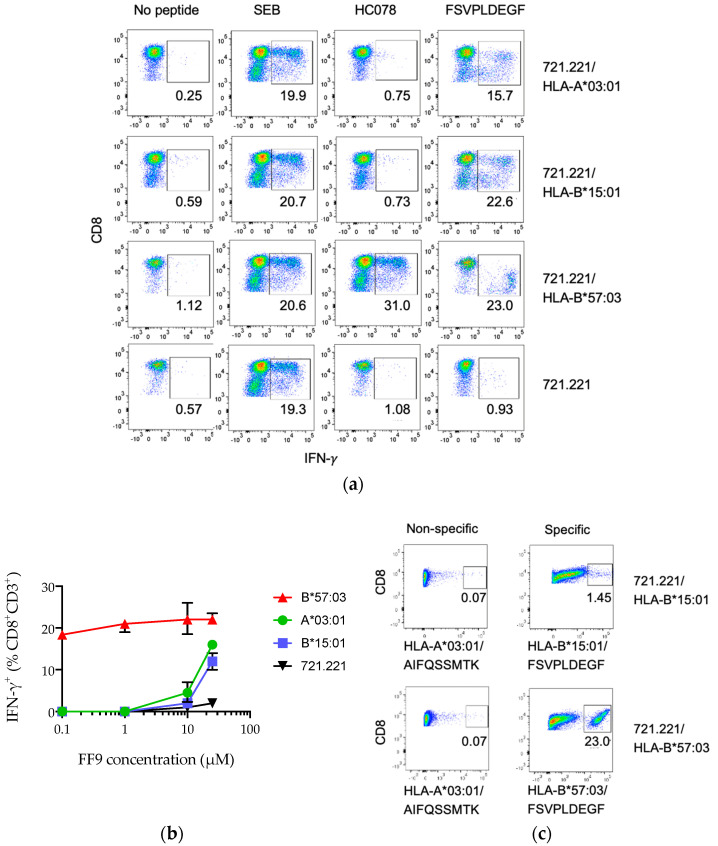
HC078-expanded STCL derived from volunteer 873 recognized FSVPLDECF (FF9) in the context of three HLA alleles. (**a**) PBMC of volunteer 873 expanded using HC078 YFSVPLDEGFRKYTA for 10 days showed different IFN-γ staining patterns of CD8^+^CD3^+^ cells when stimulated with 721,221 cells stably transfected with HLA-A*03:01, B*15:01 and B*57:03 (right) pulsed with either HC078 or FSVPLDECF peptides (above). Staphylococcal enterotoxin B (SEB) was used as a non-specific positive control. (**b**) FF9 titrations revealed strong stimulation using HLA-B*57:03 and weak interactions with HLA-A*03:01- and HLA-B*15:01-restricted cells. (**c**) HC078 STCL was stimulated with HLA-transfected 721.221 cells pulsed with FSVPLDECF (right) and CD8^+^CD3^+^ cells were assessed for interaction with either relevant (specific) or irrelevant (non-specific) HLA/peptide tetramers (below) For (**a**) and (**c**), the percentage of stained cells is inserted below the gated population.

Vaccine recipient 873 (A*03:01, A*26:01; B*15:01, B*57:03, C*04:01, C*07:01) responded to peptide HC078 YFSVPLDEGFRKYTA. Using HC078-expanded STCL, we found the strongest reactive peptide to be FSVPLDEGF (FF9), while a cluster of longer HC078 derivatives YT14, YY13, YK12, YR11, and YF10 also induced a functional T-cell response (Figure S4a). Using HLA-transfected 721.221 cells, FF9 presented by HLA-B*57:03 stimulated strongly effector cells. At the same time, HLA-A*03:01 and HLA-B*15:01-expressing cells loaded with FF9 also delivered although weaker, but a definite positive IFN-γ production signal (Figure 4a). This was further supported by peptide titration experiments (Figure 4b) and HLA/peptide tetramer reactivity for HLA-B*57:03/FF9 and HLA-B*15:01/FF9 (an HLA-A*03:01/FF9 tetramer was not available) (Figure 4c). While a potentially CD3^+^CD8^+^CD57^+^ NKT cell-mediated signal was detected within the HC078 STCL FF9-responsive population, a definite “classical” CD8^+^ T-cell component of the IFN-γ production through all restricting HLA molecules was observed (Figure S4b).

### 3.5. Heterogeneity of Vaccine-Elicited T-Cell Responses Restricted through HLA-A*02:01

**Figure 5:**
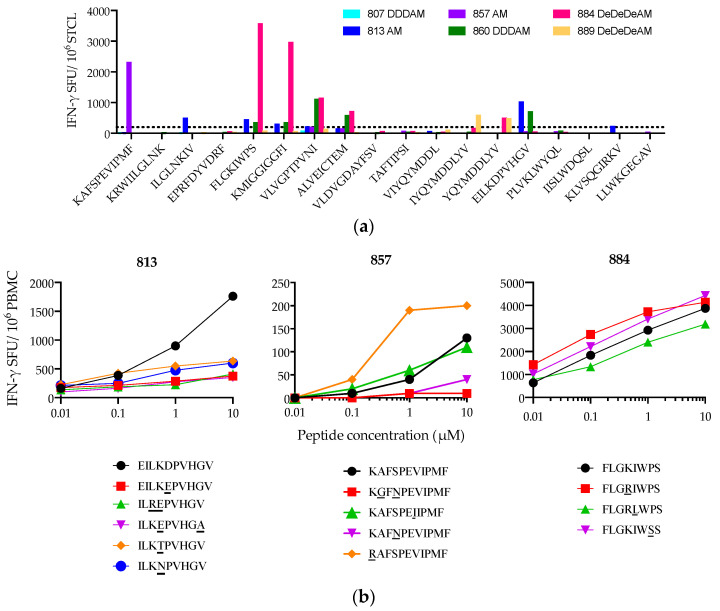
Heterogeneity in HLA-A*02:01-restricted T-cell responses. (**a**) HLA-A*02:01-positive vaccine recipients were tested for recognition of reportedly HLA-A*02:01-restricted epitopes present in HIVconsv. Their PBMC were expanded with a pool of corresponding parental 15-mers peptides containing the tested epitopes and assessed in an IFN-γ ELISPOT assay. Dotted line indicates the background frequency corresponding to 5 SFU per well. (**b**) Titration of epitope variants tested for induction of IFN-γ production by STCL.

Next, we assessed the heterogeneity of epitopes targeted in the context of a common allele HLA-A*02:01 and to what extend differences in the host genetic background impacted responses on this common allele. To this end, we tested 18 epitopes reported in the National Laboratory HIV Molecular Immunology Database (LANL-HMID) to be restricted by HLA-A2/A*02:01 solely or as well as by other HLAs [68,69] and having at least 2 out of 3 anchor residues at P2, P6 and P9 of 9-mer epitopes of the HLA-A*02:01-binding motif [21,70]. Six HLA-A*02:01-positive volunteers 807, 813, 857, 860, 884, and 889 with sufficient cryopreserved samples available were included in this analysis. Even though the optimal, truly HLA-A*02:01-presented epitope has not been defined for some of them, the six HLA-1*02:01-positive individuals recognized median (range) of 3.5 (0–6) out of these 18 tested HIVconsv epitopes (Figure 5a). Furthermore, T cells of volunteers 813, 857, and 884 also responded, albeit with variable strength, to frequently occurring natural variants of epitopes EILKDPVHGV, KAFSPEVIPMF, and FLGKIWPS, respectively (Figure 5b).

### 3.6. Suboptimal *In Vivo* Response Rescued by Index Epitope Boost

**Figure 6:**
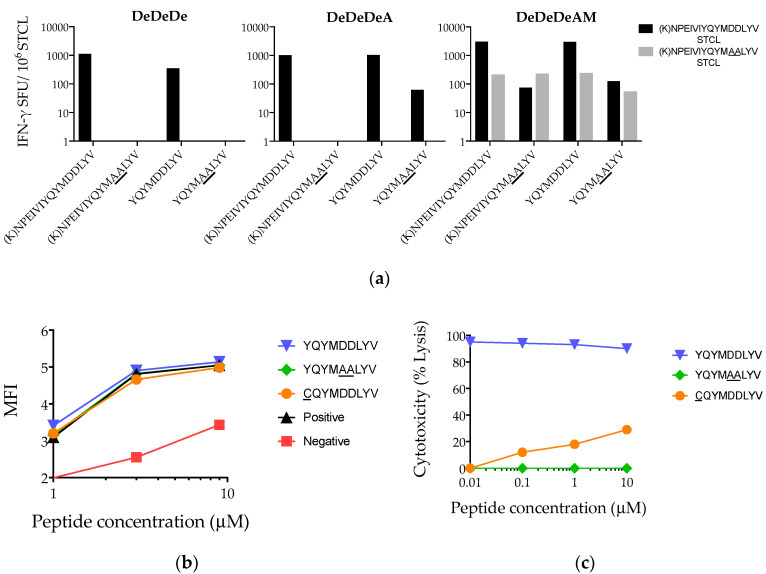
Mutant epitope prime requires index epitope boost for a functional response. (**a**) Epitope variants YQYMDDLYV and YQYMAALYV were delivered by the HIVconsv (De for electroporated DNA and M for MVA) and GRIN (Gag-RT-Integrase-Nef) (A for adenovirus) vaccines, respectively. PBMC from volunteer 884 drawn 2, 8, and 12 weeks after DeDeDe, DeDeDeA and DeDeDeAM, respectively, were expanded by either the HIVconsv (black) or GRIN (gray) 15-mer peptide variant and tested against both the parental 15-mer and 9-mer peptides in an ELISPOT assay. (**b**) Recombinant purified HLA-A*02:01 heavy chain and β_2_-microglobulin were used to test efficiency of refolding of HLA-peptide complexes in the presence of decreasing test peptide concentrations using a FACS bead assay. (**c**) Killing of peptide-pulsed HLA-A*02:01 target cells by a YQYMDDLYV-specific CD8^+^ T cell clone. CQYMDDLYV present in 1.32% of database sequences was used as a variant example of a strong binder with impaired T-cell stimulation.

To increase the safety of the GRIN immunogen, enzymatic activity of RT was inhibited by a substitution of two bulky, charged glutamic acid residues in the RT active site MDDL by smaller, hydrophobic alanines to generate MAAL [66,67]. In contrast, HIVconsv encoded the MDDL wildtype sequence. This provided a unique opportunity to assess in vivo induction of T cells to and in vitro functional recognition of a well-defined HLA-A*02:01-restricted index epitope YQYMDDLYV (YV9) [38,43,61,62,69,71,72,73] in DNA.HIVconsv and MVA.HIVconsv, and its alanine variant delivered by Ad35-GRIN. Responses were compared after DeDeDe, DeDeDeA, and DeDeDeAM in two HLA-A*02:01-positive participants 884 and 889. Only participant 884 showed a response to this region: stimulation with the mutant HC093 (K)NPEIVIYQYMAALYV peptide expanded STCL only in samples obtained after the full DeDeDeAM regimen, but not after DeDeDeA, although DeDeDeA-primed and HC093-expanded STCL recognized YQYMAALYV. Thus, Ad35-GRIN/MAAL primed T cells required a boost with the index epitope in MVA.HIVconsv/MDDL for their in vivo expansion (Figure 6a). While both YQYMDDLYV and YQYMAALYV bound HLA-A*02:01 with same efficiency (Figure 6b), the engagement of YQYMAALYV with TCR of a YQYMDDLYV-grown T-cell clone failed completely in a killing assay (Figure 6c). Another variant CQYMDDLYV present in 1.32% of HIV sequences in the LANL-HIV Sequence Database was capable of avid binding to the HLA-A*02:01 molecule and stimulating a T-cell clone killing, albeit impaired.

## 4. Discussion

Definition of CTL epitopes provides important insights into the mechanisms of host responses to HIV-1 and HIV-1′s adaptation to T-cell surveillance [14,31,74,75,76,77]. It also critically informs development of an effective HIV-1 vaccine [62,63,78,79]. In the course of this work, we characterized a number of CD8^+^ T-cell responses specific for HIV-1 conserved protein regions induced by the HIVconsv vaccines [53] in adult HIV-1/2-negative HIV-CORE 004 participants in Nairobi [60]. First in 72 trial participants, 83 HLA alleles were detected and their frequencies and those of the corresponding HLA supertypes were established. These results concurred well with previous reports on allele frequencies in this population and were distinct from Caucasian and Japanese populations [40,41,80,81,82,83,84]. It was suggested that while frequencies of HLA alleles might vary dramatically among different ethnicities and global locations, the frequency of each HLA allele supertype is remarkably constant [20,21]. Overall, we confirmed many already reported optimal CD8^+^ T-cell epitopes, for some of them identified their alternative HLA-restriction, and defined 4 novel HLA-peptide pairs, namely HLA-B*53:01/SPIETVPVK, HLA-B*07:05/SPAIFQSSMTK, HLA-B*53:01/SPAIFQSSM, and HLA-B*07:05/SPAIFQSSM [85]. These pairs represent additions to the cumulative knowledge of well-defined epitopes in this geographic region.

Volunteer 873 presented epitope FSVPLDEGF (FF9) by 3 different HLA molecules HLA-A*03:01, HLA-B*15:01 and HLA-B*57:03. These alleles belong to different HLA supertypes of A03, B62, and B58, respectively. A closer inspection of the main anchor residues in position P2 and P9 confirmed expected aa fitting into MHC pockets B and F for the strongest HLA-B*57:03 [86], but only one and none for the weaker binding alleles making the involvement of secondary anchors likely. In line with these observations, tetramer reactivity yielded different frequencies of FF9 specific cells for responses restricted by HLA-B*57:03 and HLA-B*15:01 (Figure 4c). Typical interpretation of these data would be that FF9 is presented by HLA-B*57:03, whereas the biological significance of weak HLA/peptide interactions remains unclear. However, sometimes even very weak protein–protein interactions may have biological consequences, such as those implicated in experimental challenge/protection by HLA-E-restricted CD8^+^ T cells [87,88,89,90,91] and CD4^+^ T-cell function [92,93]. It is of note that MHC-peptide affinity alone does not correlate with frequency of peptide recognition [94] nor necessarily with magnitude of responses [46] or functionality [18]. In addition, for stimulation through HLA-A*03:01, HLA-B*15:01 and HLA-B*57:03, 33.5%, 37.2%, and 25.8% subsets of the IFN-γ-producing cells in the HC078 STCL were CD3^+^CD8^+^CD57^+^ NKT cells (Figure S4b). The potential contribution of such a population to virus control will need to be further explored.

Six volunteers recognized median of 3.5 out of 18 tested epitopes recorded in the LANL-HMID to be presented through HLA-A*02:01/A2 (Figure 5a). Of note, the precision of the general listings in the LANL-HMID table of CTL/CD8^+^ Epitope Summary [85] might sometimes be questionable, which has led to the creation and regular updates of a more stringent “A” list of the best defined epitopes [85]. On the other hand, highly personalized epitope hierarchies, i.e., absence of responses to some, is not unexpected given the complexity of the antigen processing and presentation pathways [95,96,97] competition from different sets of other HLA molecules in each individual, different T-cell repertoires and, perhaps, even differences in co-infections/microbiome [98,99,100,101]. We also demonstrated that a T-cell response to an epitope with suboptimal affinity for the TCR(s) could be rescued by a vaccination with its index epitope (Figure 6). Several mechanisms could have contributed to this observation including a limited room for TCR cross-reactivity even for the perceived stringent HLA class I-peptide recognition [102] combined with multiple T-cell clones recruited into the response to this epitope [103], some of which were of too low frequency to be detected after the Ad35-GRIN prime alone. This may be another indication that even weak protein–protein interactions influence the final outcome and supports vaccines employing multivalent immunogens [69,104,105,106], although further investigations are required to generalize this observation.

## 5. Conclusions

In conclusion, we believe our study is a small, but significant contribution to the knowledge and understanding of likely protective CD8^+^ T-cell responses to HIV-1 infection in sub-Saharan Africa, where consequences of the HIV-1 epidemic still represent one of the major health and social challenges, and where an effective vaccine is urgently needed.

## Figures and Tables

**Table 1:** Summary of stimulatory human leukocyte antigen (HLA) class I T-cell epitopes in the Nairobi cohort.

No.	Parental	VID ^2^	Name	Shorter	Reported	Predicted ^1^	Confirmed
	Sequence			Sequence	HLA	HLA	HLA
HC014	EWDRIYKRWIILGLN	829	YN10	YKRWIILGLN	Not rep’d	B*27:03	
HC049	(K)NFPISPIETVPVKLK	831	SL10	SPIETVPVKL	B*81:01	B*81:01	
			IL9	IETVPVKL	B*40:01		
					B*53:01		
HC078	YFSVPLDEGFRKYTA	873	FF9	FSVPLDEGF	B*57:03	B*57:03	B*57:03
						B*15:01	B*15:01
						A*03:01	A*03:01
						A*26:01	
HC088	GSPAIFQSSMTKILE	830	AK9	AIFQSSMTK	A*03:01	A*03:01	A*03:01
					A*11:01		
			II9	IFQSSMTKI	Not rep’d	B*51:01	
		866	SK11	SPAIFQSSMTK	A*11:01		B*07:05 ♣
					A*11:01		
							B*53:01 ♣
			GM9	GSPAIFQSSM	Not rep’d	B*07:05	
			SM9	SPAIFQSSM	B7	B*07:05	B*07:05 ♣
						B*53:01	B*53:01 ♣
						C*04:02	
HC164	VQMAVFIHNFKRKGGI	830	AR9	AVFIHNFKR	A*03:01	A*03:01	
						A*74:01	
			MR10	MAVFIHNFKR	Not rep’d	A*03:01	
						A*66:01	
						A*68:01	
			MK9	MAVFIHNFK	A*03:01	A*03:01	
						A*74:01	
						B*51:01	
			VR8	VFIHNFKR	Not rep’d	A*66:01	
						A*68:01	
		889	MR10	MAVFIHNFKR	Not rep’d		
			MK9	MAVFIHNFK	Not rep’d	C*02:02	
			VR8	VFIHNFKR	Not rep’d		
HC176	VVPRRKAKIIRDYGK	831	KK10	KAKIIRDYGK	Not rep’d		
		889	KK8	KIIRDYGK	Not rep’d		
			KY8	KAKIIRDY	Not rep’d		
HC177	RKAKIIRDYGKQMAG	810	RK11	RKAKIIRDYGK	Not rep’d		
			RY9	RKAKIIRDY	B*15:03	B*15:03	

^1^ Epitopes were predicted using the LANL-HMID Immune Epitope Database (IEDB). ^2^ VID—volunteer identification. Please refer to Table S1 for volunteers’ HLAs. ♣—Previously unreported candidates for LANL-HMID “A” list.

## Supplementary Materials

The following are available online at https://www.mdpi.com/2076-393X/8/2/260/s1, Figure S1: Gating strategy for determination of HLA class I-restriction following peptide-specific restimulation, Figure S2: Responses to HC014 EWDR-IYKRWIILGLN (Gag 261–271), Figure S3: Responses to HC049 (K)NFPISPIETVPVKLK / HC050 SPIETVPVKLKPGMD (Pol 158–171), Figure S4: Responses to HC078 YFSVPLDEGFRKYTA (Pol 270–284, Figure S5. Responses to HC088 GSPAIFQSSMTKILE (Pol 311–325), Figure S6. Responses to HC164 VQMAVFIHNFKRKGGI (Pol 891–905), Figure S7: Responses to HC176 VVPRRKAKIIRDYGK (Pol 974–988), Figure S8: Responses to HC177 RKAKIIRDYGKQMAG (Pol 978–992), Table S1: HIV-1/2-negative vaccine recipients in HIV-CORE 004 used for this study, their tissue types, regimens they received, and their peak IFN-γ ELISPOT assay responses to the conserved regions of the HIVconsv immunogen induced by vaccination.
