# Supplementary material for: Specificity of CD8+ T-Cell Responses Following Vaccination with Conserved Regions of HIV-1 in Nairobi, Kenya
Source: Vaccines (Basel). 2020 May 29;8(2):260. doi: 10.3390/vaccines8020260 (PMC7349992; doi:10.3390/vaccines8020260)
Supplement: Supplementary file 1 [file vaccines-08-00260-s001.pdf]

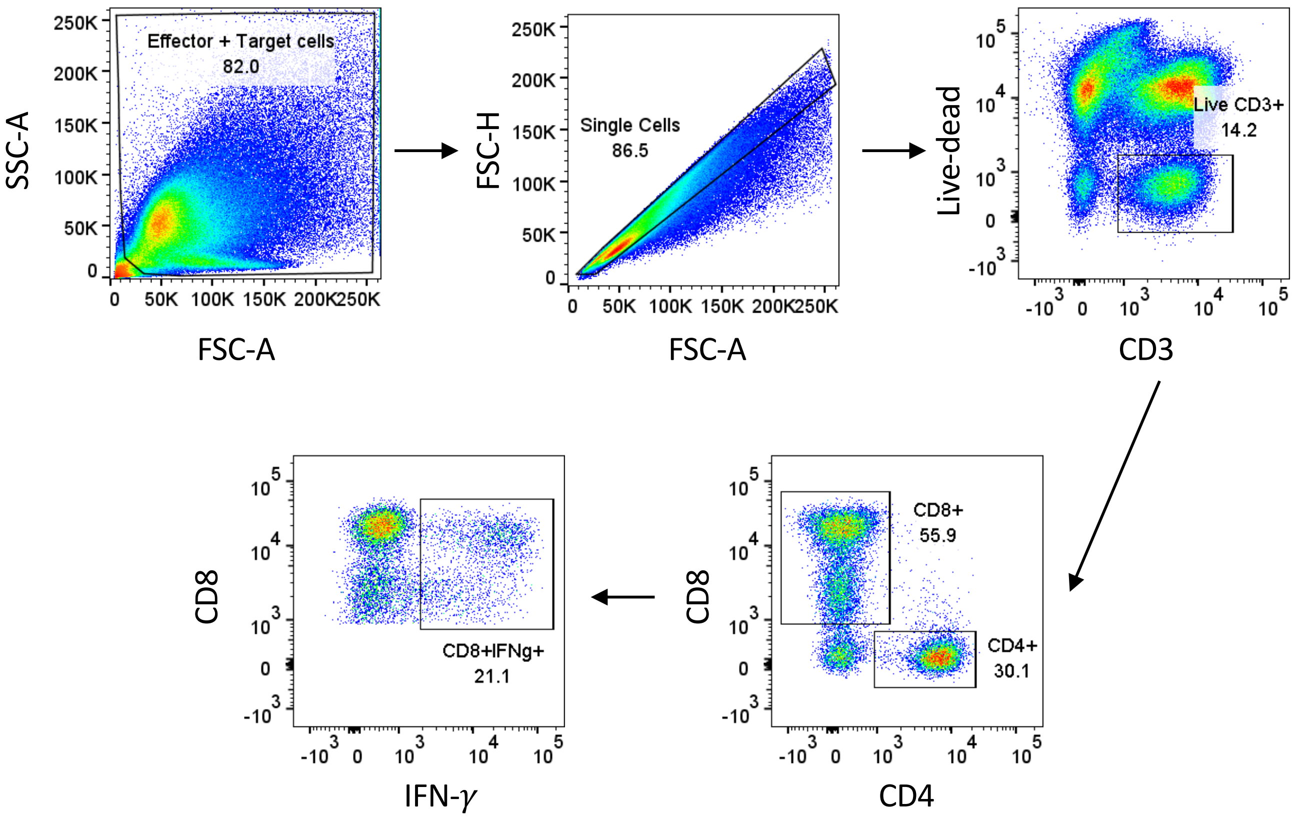

Figure S1. Gating strategy for determination of HLA class I-restriction following peptide-specific restimulation.

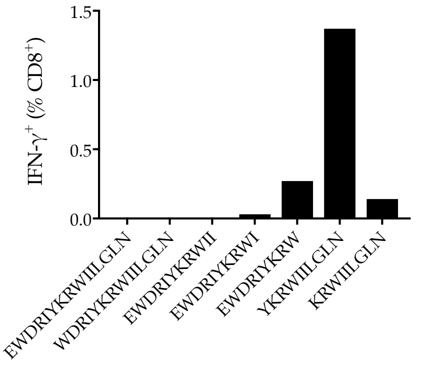

Figure S2. Responses to HC014 EWDR-IYKRWIILGLN (Gag 261-271). This is a junctional peptide spanning two adjacent regions as indicated by the hyphen, which was recognized by volunteer 829 (A*02:01, A*68:02, B*07:02, B*27:03, C*02:02, C*07:02). Using truncated peptides indicated YKRWIILGLN (YN10) as the optimum epitope, which is shared with HC015 and fully derived from HIV-1 Gag because it does not span the junction. This is not a published epitope for any of the 829's alleles and is predicted to bind HLA-B*27:03.

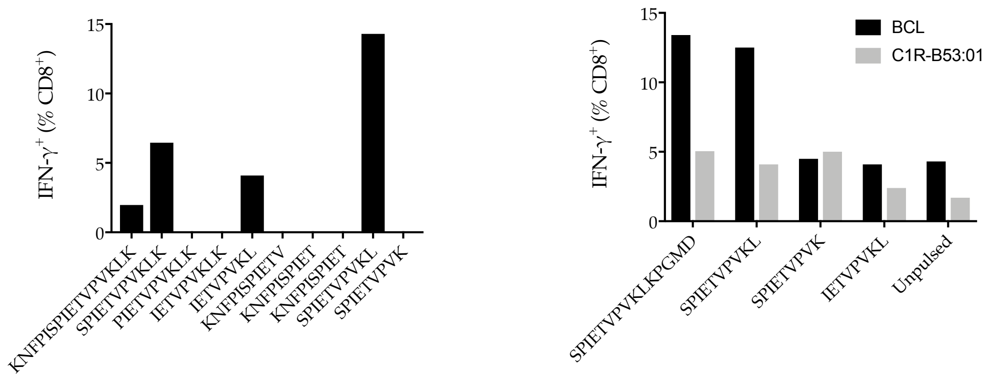

(a) (b)

Figure S3. Responses to HC049 (K)NFPISPIETVPVKLK / HC050 SPIETVPVKLKPGMD (Pol 158-171). Volunteer 831 (A*26:01, A*68:02; B*53:01, B*81:01, C*04:01) recognized peptides HC049 (N-terminal K was added to improve solubility) and HC050. Three possible shorter epitopes SPIETVPVKL (SL10), SPIETVPVK (SK9) and IETVPVKL (IL8) are predicted by EIDB. Of these, SL10 is predicted to bind HLA-B*81:01, provided a strong stimulus for HC049 STCL either just added to the SCTL (a) or pulsed onto autologous B-LCL (b) and was reported previously in a South African HIV-1+ cohort [1]. Epitope SK9 is predicted to bind HLA-B*53:01 and was confirmed using HLA-B*53:01-transfected C1R cells by providing a 3-fold increased stimulation relative to unpulsed-cell background (b). IL8 is a published epitope restricted through HLA-B*53:01 [2], but showed only weak activity using the C1R/HLA-B*53:01 cells (b).

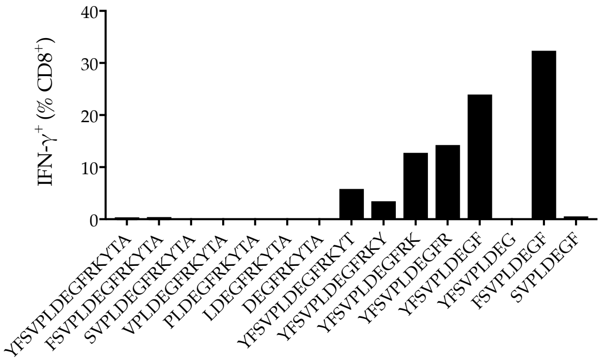

(a)

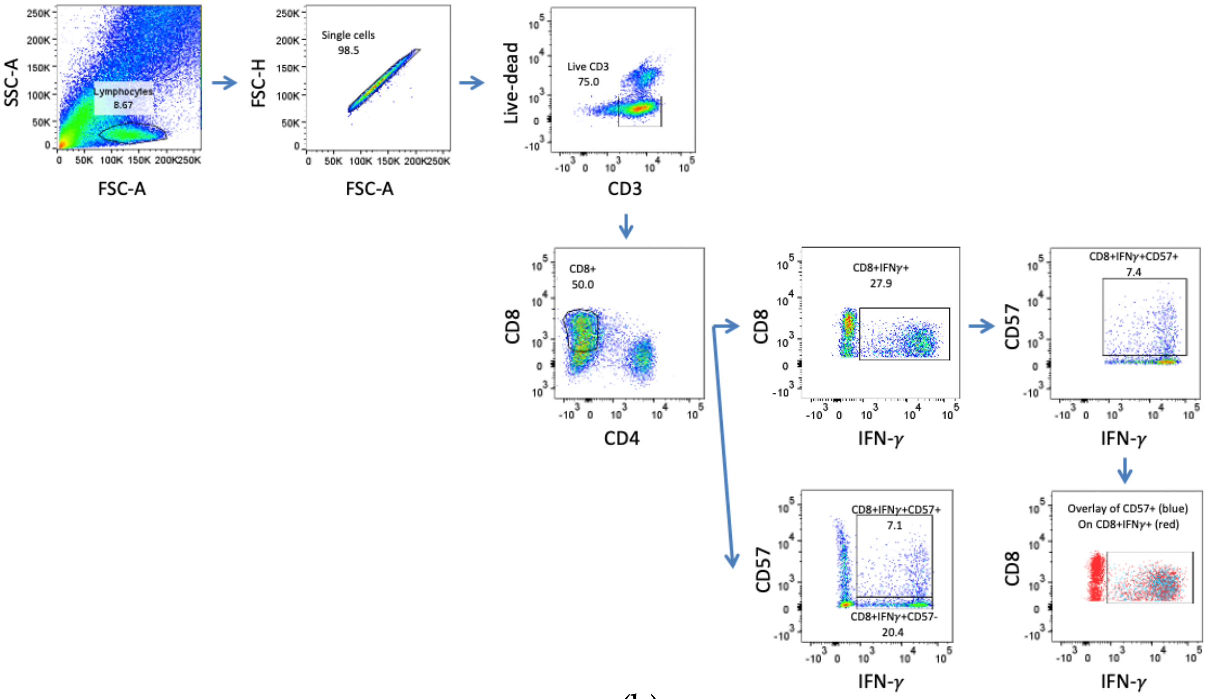

(b)

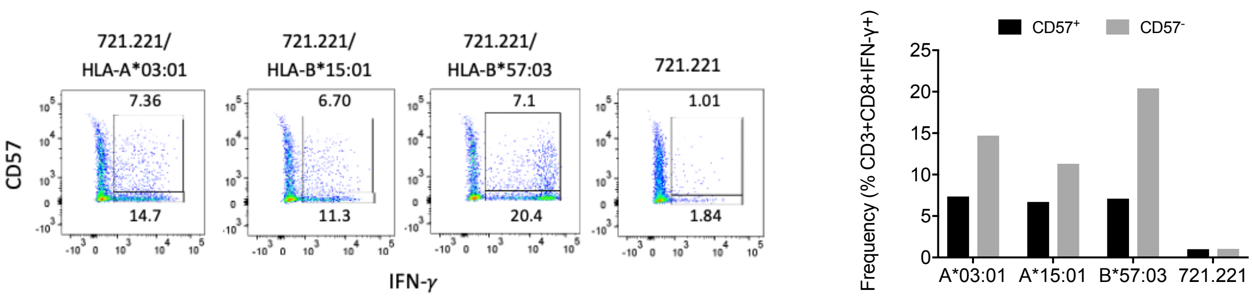

(c)

Figure S4. Continued on the next page.

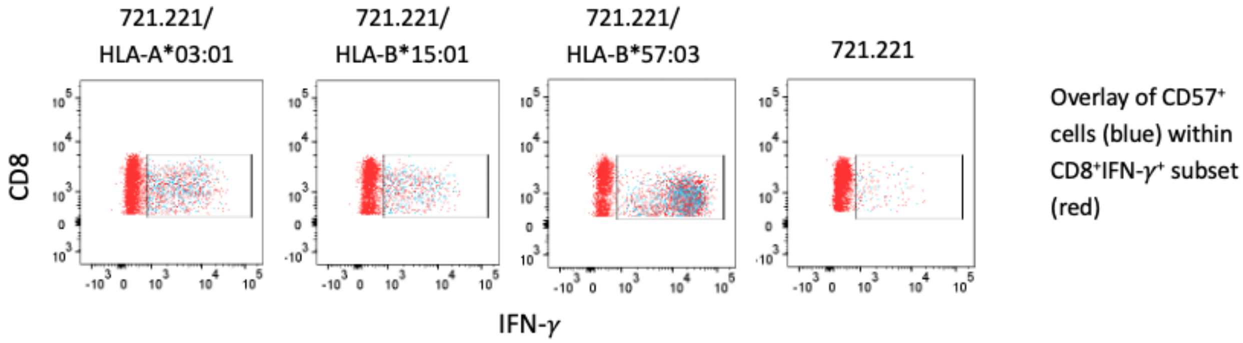

(d)

Figure S4. Responses to HC078 YFSVPLDEGFRKYTA (Pol 270-284). HC078-expanded PBMC of volunteer 873 (A*03:01, A*26:01; B*15:01, B*57:03, C*04:01, C*07:01). Studies with truncated peptides suggested FSVPLDEGF (FF9) to be the most likely epitope candidate (a). The contribution by NKT cells within the HC078 SCTL response was assessed by stimulation of SCTL with FF9-pulsed (25 μM) 721.221 cells transfected with individual HLAs using the CD57 marker. The gating strategy (b), contributing percentages of $CD8^+$ NKT cells (c) and an overlay of NKT cells on the $CD3^+CD8^+$ cells (d) are shown.

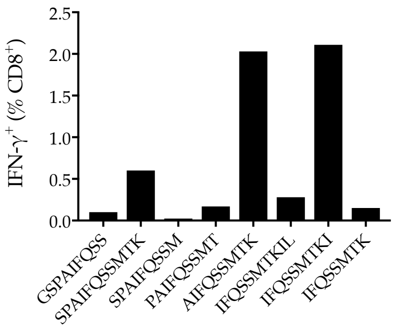

(a)

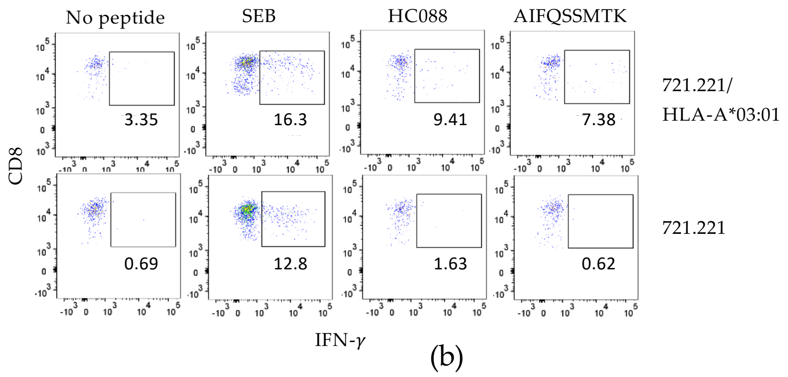

(b)

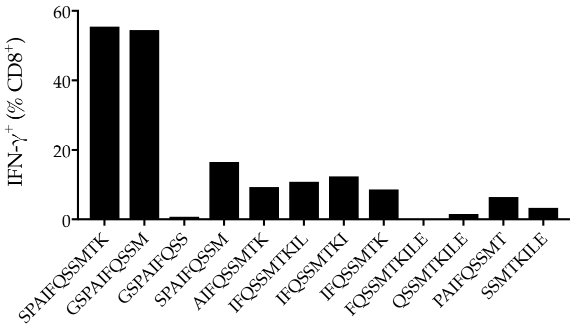

(c)

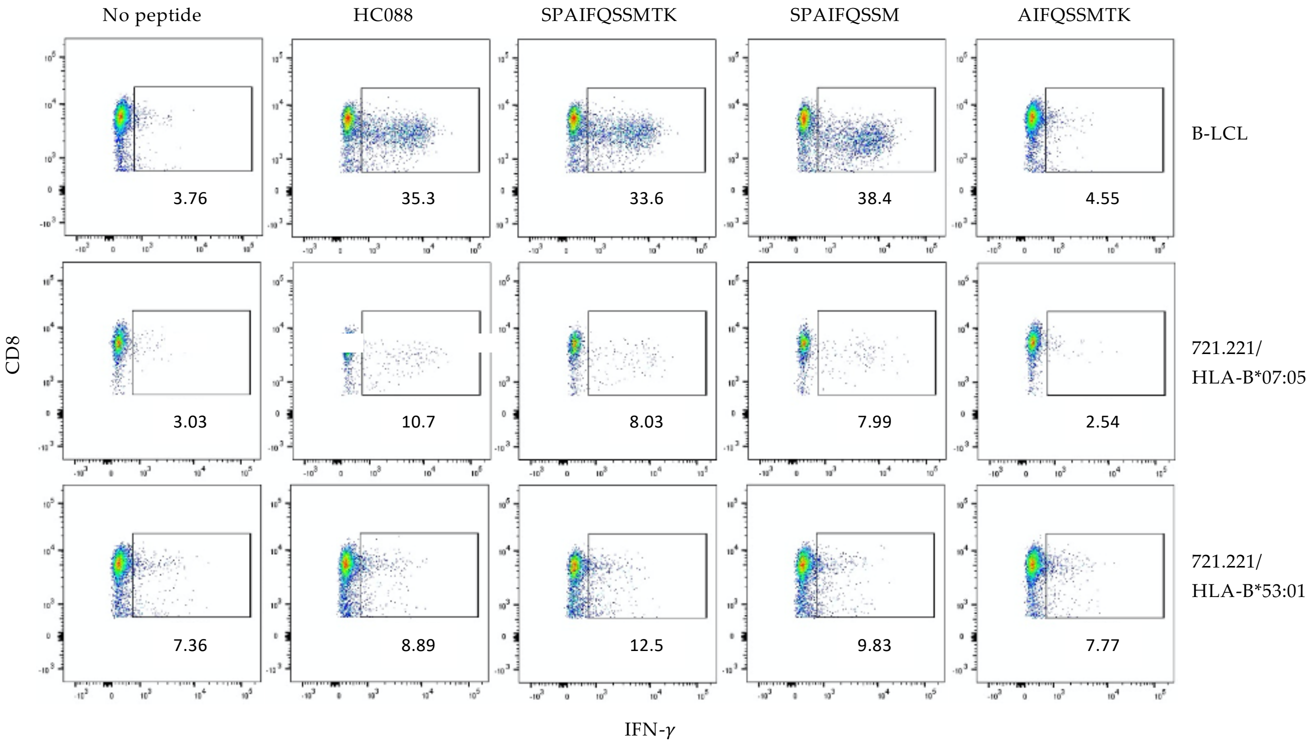

(d)

Figure S5. Continued on the next page.

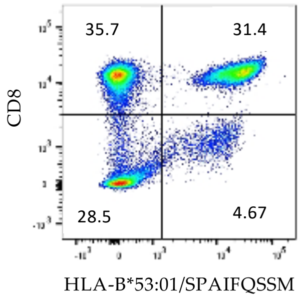

(e)

Figure S5. Responses to HC088 GSPAIFQSSMTKILE (Pol 311–325). Two volunteers responded to HC088. For subject 830 (A*03:01, A*74:01, B*08:01, B*51:01, C*04:01, C*07:01), two epitopes AIFQSSMTK (AK9) and IFQSSMTKI (II9) were suggested by the ICS assay (a). AK9 is a well reported RT epitope recognized through HLA-A*03:01 and other A03 supertype alleles [3,4]. This was confirmed using HLA-A*03:01-transfected 721.221 cell line (b). Epitope II9 is reported and is predicted to bind to 830's HLA-B*51:01. For participant 866 (A*26:01, A*34:02; B*07:05, B*53:01, C*04:01, C*08:02), a number of different potential epitopes across HC088 were detected, of which the highest IFN-γ responses were stimulated by peptides SPAIFQSSMTK (SK11) and GSPAIFQSSM (GM9) (c). We previously published SK11 restriction through HLA-B*07:02 [5], but there are no reports for any of the 866's alleles. While there is no prediction for SK11, GM9 is predicted to bind to HLA-B*07:05. SPAIFQSSM (SM9) is predicted to bind strongly HLA-B*07:05 and also HLA-B*53:01 and C*04:02. Using transfected cell lines, stimulation was found for SK11 and SM9 using HLA-B*07:05 and B*53:01 cell lines (d). HC088 STCL reacted with the HLA-B53:01/SM9 tetramer (e).

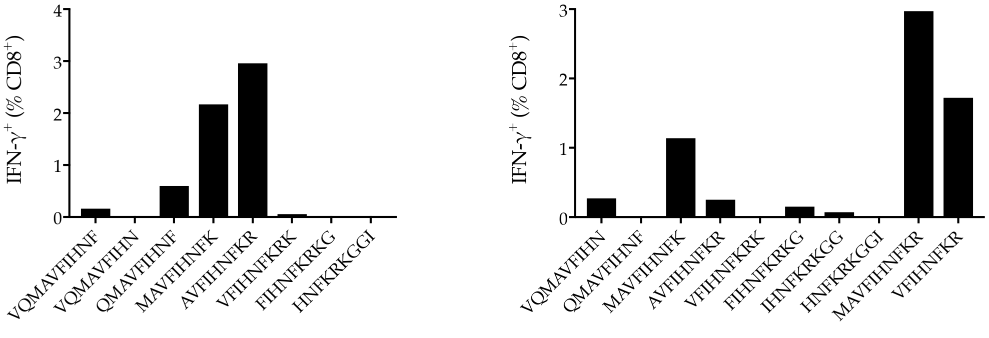

(a) (b)

Figure S6. Responses to HC164 VQMAVFIHNFKRKGGI (Pol 891-905). ICS assay in volunteer 830 (A*03:01, A*74:01; B*08:01, B*51:01, C*04:01, C*07:01) using truncated peptides suggested AVFIHNFKR (AR9) and MAVFIHNFK (MK9) to be epitopes recognized by 830 (a). Epitope AR9 was previously reported as restricted through HLA-A*03:01 [6] and is also predicted as binding to HLA-A*03:01 and HLA-A*A74:01, however, HLA-A*03:01 transfected cell line did not confirm this for 830. MK9 was reported to be restricted through HLA-A*03:01 [7] and is a predicted epitope for this allele as well as HLA-A*74:01 and HLA-B*51:01. For volunteer 889 (A*01:01, A*02:01, B*15:03, B*27:26, C*02:02, C*02:01), MAVFIHNFKR (MR10) was indicated as the epitope and to a lesser extent VFIHNFKR (VR8) and MK9 (b). There are no data published for these alleles and epitopes. The best prediction is MK9 binding to HLA-C*02:02.

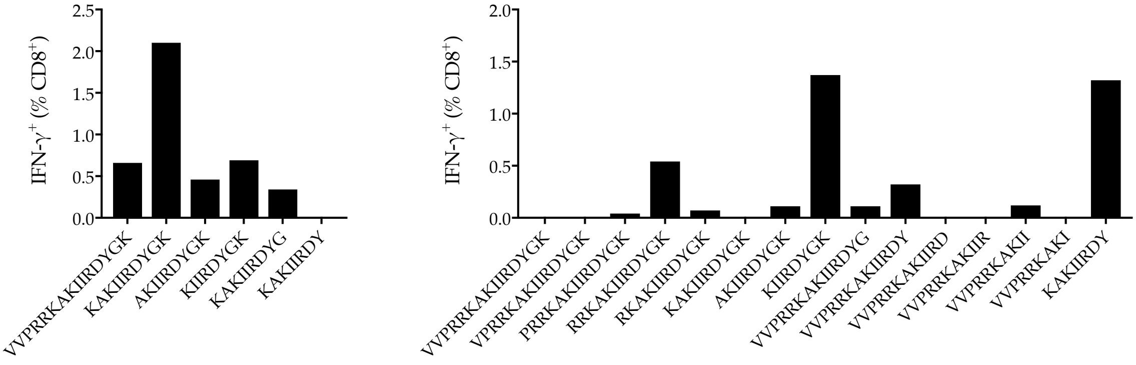

(a) (b)

Figure S7. Responses to HC176 VVPRRKAKIIRDYGK (Pol 974-988). HC176 was stimulatory in volunteer 831 (A*26:01, A*68:02; B*53:01, B*81:01, C*04:01). Shorter peptides suggested KAKIIRDYGK (KK10) as the most likely epitope (a), which has not been published and is not predicted for any of subject's alleles. For 889 (A*01:01, A*02:01, B*15:03, B*27:26, C*02:02, C*02:01), two optimal epitopes, KIIRDYGK (KK8) and KAKIIRDY (KY8) were readily detected (b), neither of which is reported or predicted. The 9-mer RKAKIIRDY peptide is a published epitope restricted through HLA-B*15:03 [8] and is also predicted to bind HLA-B*15:03 and B*27:26.

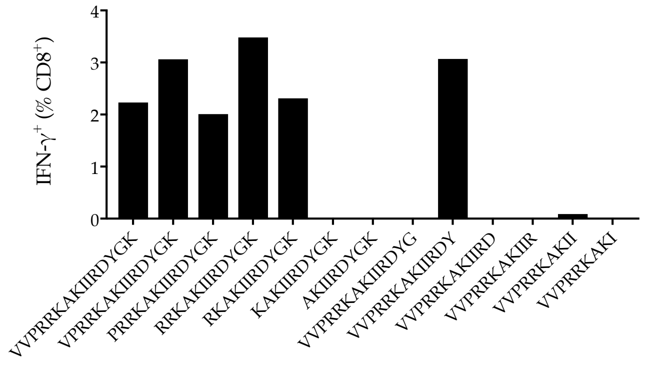

(a)

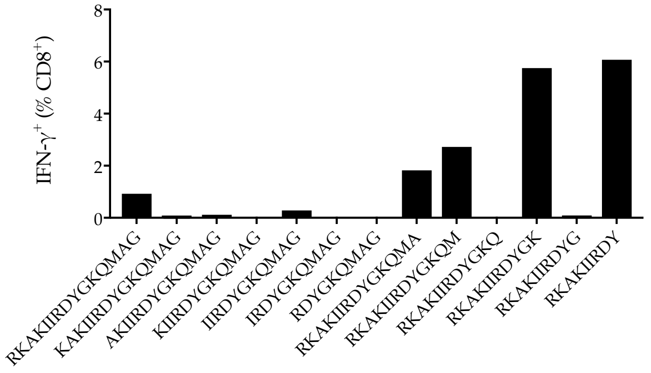

(b)

Figure S8. Responses to HC177 RKAKIIRDYGKQMAG (Pol 978-992). Volunteer 810 (A*02:01, A*03:01, B*15:03, B*42:01, C*02:01, C*17:01) responded to both 15-mers HC176 and HC177 in an IFN-γ ELISPOT assay. Using truncated peptides across both parental peptides indicated RKAKIIRDYGK (RK11) and RKAKIIRDY (RY9) as possible epitopes. There is nothing reported or predicted for RK11, but RY9 was reported as restricted through B*15:03 [9] and is also a predicted epitope for this HLA, which was the most frequent B allele in the HIV-CORE 004 cohort.

Table S1. HIV-1/2-negative vaccine recipients in HIV-CORE 004 used for this study, their tissue types, regimens they received, and their peak IFN-γ ELISPOT assay responses to the conserved. regions of the HIVconsv immunogen induced by vaccination.

VID	HLA class I			Regimen[1]	Peak IFN-γ ELISPOT (SFU/10^6 PBMC)[2]
	HLA-A	HLA-B	HLA-C		
810	*02:01 *03:01	*15:03 *42:01	*02:01 *17:01	DeDeDeAM	1781
813	*02:01 *68:02	*07:02 *53:01	*04:01 *15:05	AM	1163
823	*66:01 *74:01	*45:01 *58:02	*06:02 *06:02	DeDeDeAM	2017
829	*02:01 *68:02	*07:02 *27:03	*02:02 *07:02	DeDeDeAM	5300
830	*03:01 *74:01	*08:01 *51:01	*04:02 *07:01	DeDeDeAM	3890
831	*26:01 *68:02	*53:01 *81:01	*04:01 *04:01	DeDeDeAM	7280
857	*02:01 *23:01	*53:01 *57:03	*04:01 *08:02	AM	1893
860	*02:01 *30:01	*42:01 *51:01	*16:01 *17:01	DDDAM	1713
861	*02:05 *36:01	*27:03 *53:01	*02:02 *04:01	DeDeDeAM	5145
866	*26:01 *34:02	*07:05 *53:01	*04:01 *08:02	DDDAM	1693
867	*01:09 *29:02	*15:17 *44:15	*04:07 *17:01	DeDeDeAM	1315
873	*03:01 *26:01	*15:01 *57:03	*04:01 *07:01	DDDAM	6567
877	*23:01 *33:01	*15:10 *45:01	*03:04 *06:02	DeDeDeAM	4413
884	*02:01 *29:02	*42:01 *53:01	*06:02 *17:01	DeDeDeAM	4020
889	*01:01 *02:01	*15:03 *27:26	*02:02 *02:01	DeDeDeAM	9613
896	*23:01 *66:01	*08:01 *44:03	*04:01 *07:01	DeDeDeAM	1902
8106	*02:02 *29:02	*42:01 *57:03	*07:01 *17:01	DeDeDeAM	6310

[1] Please refer to Figure 1 for regimens and doses.

[2] HIVconsv-specific spot-forming units (SFU; can be single or a cluster of cells) per 10^6 of PBMC.

REFERENCES

1. Kiepiela, P., A.J. Leslie, I. Honeyborne, D. Ramduth, C. Thobakgale, S. Chetty, P. Rathnavalu, C. Moore, K.J. Pfafferott, L. Hilton, P. Zimbwa, S. Moore, T. Allen, C. Brander, M.M. Addo, M. Altfeld, I. James, S. Mallal, M. Bunce, L.D. Barber, J. Szinger, C. Day, P. Klenerman, J. Mullins, B. Korber, H.M. Coovadia, B.D. WalkerP.J. Goulder, *Dominant influence of HLA-B in mediating the potential co-evolution of HIV and HLA.* Nature, 2004. **432**(7018): p. 769-75. nature03113 [pii] 10.1038/nature03113
2. Pereyra, F., D. Heckerman, J.M. Carlson, C. Kadie, D.Z. Soghoian, D. Karel, A. Goldenthal, O.B. Davis, C.E. DeZiel, T. Lin, J. Peng, A. Piechocka, M. CarringtonB.D. Walker, *HIV control is mediated in part by CD8+ T-cell targeting of specific epitopes.* J Virol, 2014. **88**(22): p. 12937-48. 10.1128/JVI.01004-14
3. Menendez-Arias, L., A. MasE. Domingo, *Cytotoxic T-lymphocyte responses to HIV-1 reverse transcriptase (review).* Viral Immunol, 1998. **11**(4): p. 167-81. 10.1089/vim.1998.11.167.
4. Threlkeld, S.C., P.A. Wentworth, S.A. Kalams, B.M. Wilkes, D.J. Ruhl, E. Keogh, J. Sidney, S. Southwood, B.D. WalkerA. Sette, *Degenerate and promiscuous recognition by CTL of peptides presented by the MHC class I A3-like superfamily: implications for vaccine development.* J Immunol, 1997. **159**(4): p. 1648-57.
5. Borthwick, N., Z. Lin, T. Akahoshi, A. Llano, S. Silva-Arrieta, T. Ahmed, L. Dorrell, C. Brander, H. Murakoshi, M. TakiguchiT. Hanke, *Novel, in-natural-infection subdominant HIV-1 CD8+ T-cell epitopes revealed in human recipients of conserved-region T-cell vaccines.* PLoS One, 2017. **12**(4): p. e0176418. 10.1371/journal.pone.0176418.
6. Reche, P.A., D.B. Keskin, R.E. Hussey, P. Ancuta, D. GabuzdaE.L. Reinherz, *Elicitation from virus-naive individuals of cytotoxic T lymphocytes directed against conserved HIV-1 epitopes.* Med Immunol, 2006. **5**: p. 1. 10.1186/1476-9433-5-1
7. Propato, A., E. Schiaffella, E. Vicenzi, V. Francavilla, L. Baloni, M. Paroli, L. Finocchi, N. Tanigaki, S. Ghezzi, R. Ferrara, R. Chesnut, B. Livingston, A. Sette, R. Paganelli, F. Aiuti, G. PoliV. Barnaba, *Spreading of HIV-specific CD8+ T-cell repertoire in long-term nonprogressors and its role in the control of viral load and disease activity.* Hum Immunol, 2001. **62**(6): p. 561-76. 10.1016/s0198-8859(01)00245-2
8. Cao, J., J. McNevin, U. MalhotraM.J. McElrath, *Evolution of CD8+ T cell immunity and viral escape following acute HIV-1 infection.* J Immunol, 2003. **171**(7): p. 3837-46. 10.4049/jimmunol.171.7.3837
9. Frahm, N., P. Kiepiela, S. Adams, C.H. Linde, H.S. Hewitt, K. Sango, M.E. Feeney, M.M. Addo, M. Lichterfeld, M.P. Lahaie, E. Pae, A.G. Wurcel, T. Roach, M.A. St John, M. Altfeld, F.M. Marincola, C. Moore, S. Mallal, M. Carrington, D. Heckerman, T.M. Allen, J.I. Mullins, B.T. Korber, P.J. Goulder, B.D. WalkerC. Brander, *Control of human immunodeficiency virus replication by cytotoxic T lymphocytes targeting subdominant epitopes.* Nat Immunol, 2006. **7**(2): p. 173-8.
